# What is the appropriate duration of adjuvant imatinib mesylate treatment for primary gastrointestinal stromal tumors classified according to the strict definition of tumor rupture?

**DOI:** 10.1097/MD.0000000000014177

**Published:** 2019-01-18

**Authors:** Jun Lu, Yun Dai, Hua-Long Zheng, Jian-Wei Xie, Jia-Bin Wang, Jian-Xian Lin, Qi-Yue Chen, Long-Long Cao, Mi Lin, Ru-Hong Tu, Ze-Ning Huang, Ju-Li Lin, Ping Li, Chang-Ming Huang, Chao-Hui Zheng

**Affiliations:** a Department of Gastric Surgery, Fujian Medical University Union Hospital; b Department of General Surgery, Fujian Medical University Union Hospital; c Key Laboratory of Ministry of Education of Gastrointestinal Cancer, Fujian Medical University, Fuzhou, Fujian, China.

**Keywords:** GIST, imatinib mesylate, prognosis, tumor rupture

## Abstract

In gastrointestinal stromal tumors (GISTs), rupture is a high-risk feature; however, “tumor rupture” is inconsistently defined, and its prognostic value remains controversial.

Six hundred ninety-one patients undergoing surgery for primary nonmetastatic GISTs from 2003 to 2015 at our institution were enrolled. The strict definitions of “tumor rupture” according to the Kinki GIST Study Group (KGSG) were used.

The median follow-up time was 64 months. The 5-year recurrence-free survival (RFS) and overall survival (OS) rates in the entire group were 79.3% and 84.1%, respectively. According to the KGSG's definition, tumor rupture occurred only in 24 (3.5%) of 691 patients. For all 691 patients, multivariable analysis showed that tumor rupture, according to KGSG's definition, is one of the independently prognostic factors for both RFS and OS. Twenty-four patients with tumor rupture were further analyzed. Receiving IM for more than 3 years was significantly associated with improved RFS and OS in GISTs patients with tumor rupture.

Tumor rupture according to KGSG's definition was an independent predictive factor associated with GIST patient prognosis. More importantly, for GISTs with tumor rupture according to the KGSG's strict definition, receiving IM treatment for ≥3 years should be considered.

## Abstract 2

Supplemental Digital Content is available in the text

## 1 Introduction

Gastrointestinal stromal tumor (GIST) is the most common mesenchymal neoplasm of the alimentary tract. Complete surgical resection is the only potentially permanent cure for localized, primary GISTs; however, disease relapse may occur in approximately 40% of patients even after complete resection.^[1]^ Tumor rupture, either spontaneous or iatrogenic, used as a factor in the risk stratification improved the sensitivity for predicting the recurrence of GISTs after resection.^[1–3]^ However, whether rupture is an independent prognostic risk factor is still controversial^[4]^; it has failed to remain as a prognostic factor in several large series.^[5–8]^ This uncertainty as to its independent prognostic significance could be explained by the inconsistent definition of tumor rupture.^[1,5,9–11]^

Tumor rupture is generally regarded as an indication for adjuvant imatinib (IM) treatment.^[2]^ Based on 3 randomized studies, trial Z9001 by the American College of Surgeons Oncology Group (ACOSOG),^[12]^ randomized trial 62024 sponsored by the European Organization for Research and Treatment of Cancer,^[13]^ and the Scandinavian Sarcoma Group (SSG) XVIII/Arbeitsgemeinschaft Internistische Onkologie (AIO) trial,^[14]^ it is currently recommended that patients with ruptured GIST require prolonged adjuvant therapy for 3 years.^[15]^ However, there has been no precise or common definition of rupture in these trials. Long-term adjuvant treatment with IM may also cause resistance, adverse effects, and result in a heavy economic burden; therefore, patient selection for appropriate duration adjuvant treatment is critically important, and a clear definition of “rupture” has significant therapeutic implications.

Recently, strict definitions of ruptured GIST were proposed by the Kinki GIST Study Group (KGSG)^[10]^; however, until now, no study has examined the appropriate duration of adjuvant IM treatment for primary GIST classified according to the strict definitions of tumor rupture.

In this study, the current strict definition of tumor rupture proposed by KGSG was verified, and the appropriate duration of receiving IM adjuvant therapy for GISTs with tumor rupture was further investigated.

## 2 Materials and methods

### 2.1 Patients

Between January 2003 and December 2015, a total of 691 patients with primary pathologically proven GISTs undergoing surgery in a large tertiary hospital (Fujian Medical University Union Hospital [FMUUH]) were collected from the prospective GIST database. The inclusion criteria were as follows: patients treated without neoadjuvant IM; patients with complete sets of IM information, clinicopathological and follow-up data; patients without distant metastasis; and patients with no other synchronous malignancy. Those who did not undergo surgery for their primary tumor; those with metastatic or recurrent GIST at the time of diagnosis; individuals whose data such as age, sex, or general tumor features were missing; or those who died within 30 days after surgery were excluded. Tumor size was defined as the maximum tumor diameter. The mitotic rates were defined as the number of mitoses per 50 high-power fields (HPF). Risk stratification was assessed according to the modified National Institutes of Health (NIH) consensus criteria.^[16]^ Surgical and histopathological reports were reviewed carefully for completeness of information on tumor rupture. Administration of adjuvant IM was decided during multidisciplinary team meetings. Patients were divided into groups (<3 years and ≥3 years) according to the time of receiving IM. The time of IM therapy was from the first time the patient took IM to the last time before patients were confirmed with relapse, as we described previously.^[17]^ The dose of IM in most of the eligible patients was 400 mg q.d. For those patients with a Grade 3/4 event such as intolerable cardiac failure and reduplicative Neutropenia, 300 mg q.d. was also considered to be competent. Informed consent was obtained from all patients. This study was reviewed and approved by the institutional review board of FMUUH.

### 2.2 Definition of tumor rupture

Tumor rupture was defined as proposed by the KGSG, which includes perforation at the tumor site, tumor fracture with blood-tinged ascites, piecemeal resection during surgery (including open biopsy), and macroscopic injuries to the pseudocapsule exposing tumor cells into the peritoneal cavity.^[10]^ The definitions of rupture proposed by KGSG were classified as tumor rupture and nonrupture. The criteria for the assessment of resection margins (R status) were as follows^[18]^: R0, no detectable residual tumor; R1, microscopic residual tumor; and R2, macroscopic residual tumor.

### 2.3 Follow-up

As we described previously,^[17]^ a regular follow-up conducted for at least 5 years. Follow-up assessment included physical examination and abdominal computed tomography (CT) every 6 months for the first 2 years and yearly thereafter. Upper gastrointestinal endoscopy was performed if clinical and/or CT abnormalities were detected during follow-up. Median follow-up was 64 (range 8–195) months.

### 2.4 Statistical analysis

Statistical analyses were performed using the independent *t* test, chi-squared test, and Fisher exact test. Recurrence-free survival (RFS) was calculated from the date of surgery until first recurrence or to the last date of follow-up if GIST had not recurred. Overall survival (OS) was calculated from the date of surgery to the date of any death or the last available follow-up. Survival was estimated using the Kaplan–Meier method and compared using the log-rank test. Multivariable analysis was conducted to identify risk factors associated with RFS and OS, using the forward stepwise Cox proportional hazards regression model. All tests were 2-sided, and a *P* value <.05 was considered statistically significant. Data analysis was performed using IBM SPSS Statistics Package, version 22.0 (IBM Corporation, Armonk, NY).

## 3 Results

### 3.1 Clinicopathological characteristics

From 2003 to 2015, 691 patients who underwent R0 or R1 surgery for primary, nonmetastatic gastric GISTs entered the study. The total cohort included 410 (59.3%) males. Median age was 56 years (range 20–82). Median tumor size was 5.6 cm (range 0.5–25.0), and median mitotic index was 3 per 50 HPF (range 0–78). According to the definition of KGSG, tumor rupture was recorded in 24 (3.5%) patients. The clinicopathological and demographic features of patients with and without defects in tumor integrity are summarized in Table 1. The subgroup distributions of patients according to the KGSG definitions of the tumor rupture are shown in Table 2.

**Table 1:**
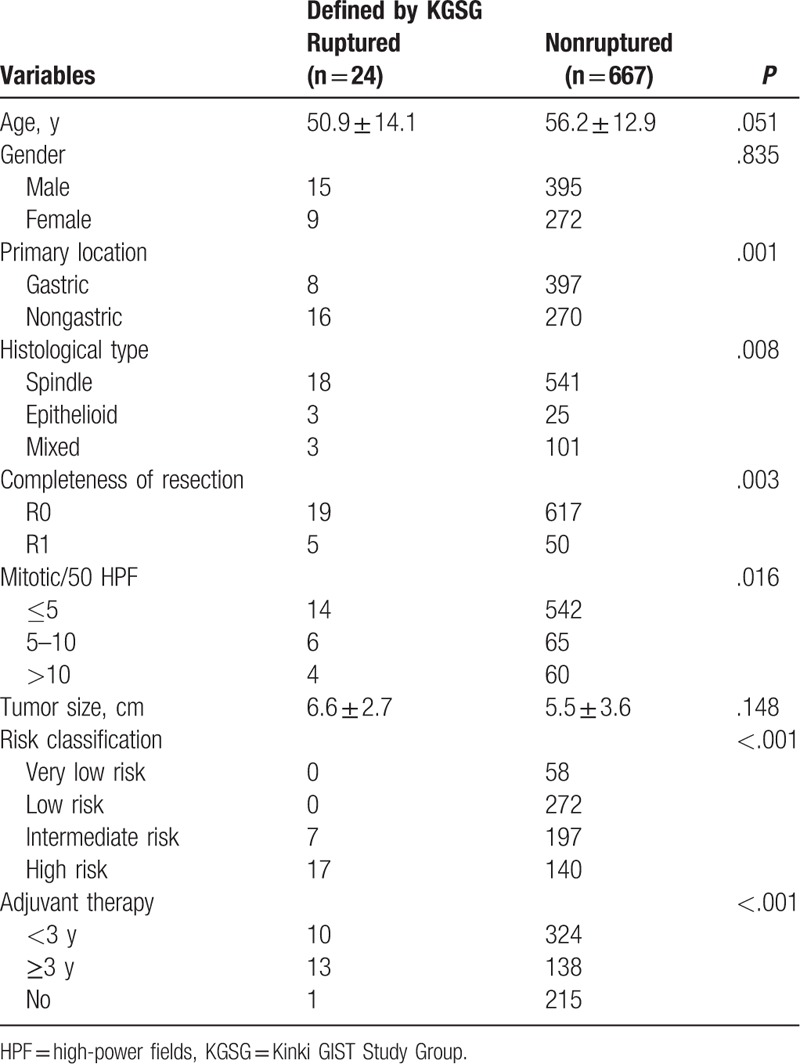
Clinicopathologic characteristics.

**Table 2:**
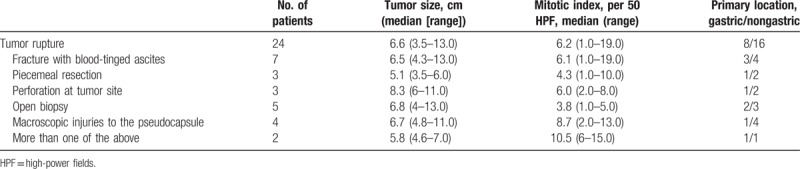
Defects of tumor integrity related to risk factors.

### 3.2 Survival after surgery for GIST patients

**Figure 1:**
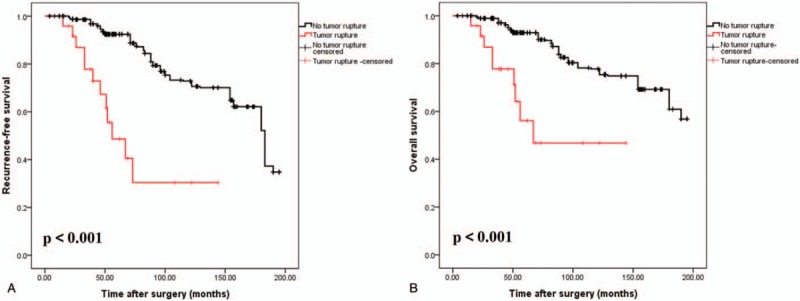
Recurrence-free (A) and overall (B) survivals after resection of GISTs in relation to tumor integrity. Tumor integrity was defined by KGSG. GIST = gastrointestinal stromal tumors, KGSG = Kinki GIST Study Group.

**Figure 2:**
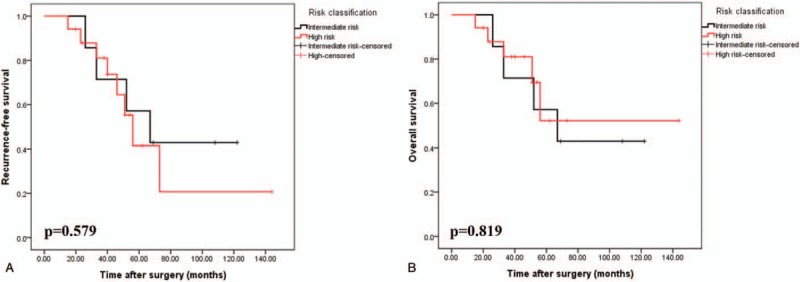
Recurrence-free (A) and overall (B) survivals after surgery for 24 patients with tumor rupture according to the KGSG definition, grouped by risk classification. KGSG = Kinki GIST Study Group.

As shown in Fig. 1, the 5-year RFS and OS results were analyzed according to the defects in tumor integrity. Based on the classification of KGSG, the 5-year RFS and OS values were 44.2% and 52.2%, respectively, for patients with tumor rupture and 92.4% and 92.9%, respectively, for patients with no tumor rupture (*P* < .001) (Fig. 1A and B). Moreover, the 5-year RFS of intermediate- and high-risk patients with tumor rupture was not significant different (57.1% vs 41.5%, *P* = .597). Similar results were found in terms of OS analysis (57.1% vs 52.1%, *P* = .819) (Fig. 2A and B).

### 3.3 Multivariable survival analysis

Multivariate analyses using the Cox proportional hazards model indicated that tumor location, tumor size, mitotic count, and rupture defined by KGSG were independent prognostic factors for RFS and that age and rupture defined by KGSG were independent prognostic factors for OS in the study cohort. It is worth noting that R1 resection was not the statistically significant factor associated with survival in multivariate analyses, which was consistent with previous study^[19]^ (Table 3). The first possible explanation for this interesting finding is that the rupture defined by KGSG has a stronger prognostic value than R1 resection. The second reason may due to the small number of cases.

**Table 3:**
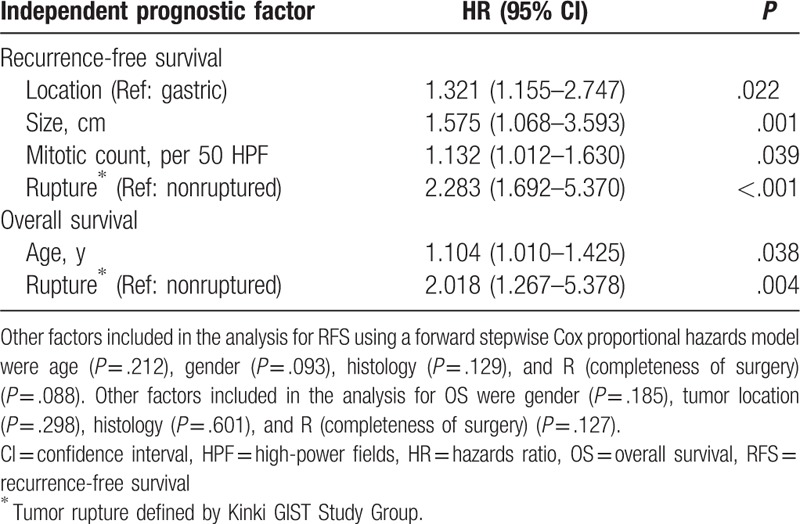
Multivariate analysis for RFS and OS.

### 3.4 Stratified analysis of IM therapeutic effects on patients with ruptured tumors GISTs according to KGSG

**Figure 3:**
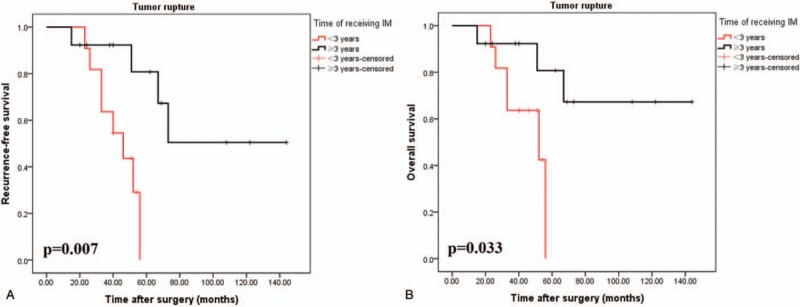
Recurrence-free (A) and overall (B) survivals after surgery for 24 patients with tumor rupture according to the KGSG definition, grouped by different durations of IM. KGSG = Kinki GIST Study Group, IM = imatinib.

The 5-year RFS rates of patients with tumor rupture in the <3 years and ≥3 years group were 29.1% and 80.8%, respectively (*P* = .007). Moreover, the 5-year OS rates of patients with tumor rupture in the <3 years and ≥3 years group were 42.4% and 80.8%, respectively (*P* = .033) (Fig. 3A and B).

## 4 Discussion

GISTs, mostly caused by mutations in the PDGFRA and KIT genes, are the most common mesenchymal tumor in the gastrointestinal tract. The true incidence of tumor rupture is speculated to be several percent in real clinical practice. Rupture was introduced as a risk factor in the modified NIH consensus criteria,^[16]^ along with the acknowledged variables of mitotic index, tumor size, and anatomical site.^[20,21]^ The Joensuu modified NIH classification combines the advantages of the NIH and Armed Forces Institute of Pathology criteria with the additional factor of rupture.^[5]^

Ruptured tumors were frequently large GISTs with a high mitotic count located in the small bowel, and rupture was also associated with GIST genotypes.^[22–24]^ One of the latest studies from Oslo University Hospital (OUH) concludes that gastric GISTs with del 557/558 have a higher risk of rupture than tumors with other mutations.^[25]^ A multicenter study from Europe suggested that GIST with tumor ruptures often have other adverse prognostic features, such as large size, a high mitotic count, and a nongastric site of origin.^[5]^ This present study showed that ruptured GIST exhibited aggressive features such as higher mitotic count and risk classification compared with minor defect or nonruptured tumors, similar to previous studies.^[10,11]^

Because the definition of rupture is not uniform, the reported incidence of rupture in GIST series varies considerably in different series, from <2% to >22%.^[1,5,7,9–11]^ One study from North America enrolled 502 GIST patients, and tumor rupture was recorded only in 7 (1.4%) patients.^[7]^ Rutkowski et al^[5]^ found that tumor rupture occurred in 46 (7%) out of the 640 cases. A recent study published by KGSG found that tumor rupture occurred in 3.2% of 665 primary GIST patients.^[10]^ Using the same KGSG definition, ruptured GIST was seen in 3.5% of primary GISTs in our series.

Prognostic factors for GIST recurrence after surgery have been widely investigated. Among these prognostic factors, tumor rupture is the most ominous and is a subjective factor clinically judged by surgeons.^[1,4,10]^ It is controversial whether tumor rupture is an independent prognostic factor.^[2,4,5,9–11,16]^ In a series of 335 GIST patients, Rutkowski and colleague identified tumor rupture as an independent prognostic factor.^[9]^ Subsequently, in results from a large cohort of patients with operable GIST published by Joensuu et al, rupture was shown to have an independent, adverse effect on prognosis.^[4]^ A stronger relationship between rupture and recurrence was demonstrated in the SSG XVIII/AIO trial.^[3]^ The sarcoma group at OUH has demonstrated that rupture is a strong and independent risk factor for recurrence, even when other established prognostic factors are taken into consideration.^[26]^ However, other large studies have failed to corroborate this outcome.^[5,7,8]^ Nishida et al^[10]^ showed that rupture remains an important prognostic factor for RFS, but not OS. The series of studies on RFS and OS in patients with and without tumor rupture are summarized in Supplementary Table 1. The uncertainty as to the independent prognostic significance of tumor rupture could be partly ascribed to inconsistent definitions and incomplete reporting.

At present, there are 2 strict criteria for tumor ruptures (Supplementary Table 2). One was proposed by the KGSG,^[10]^ and the other was proposed by the Oslo Sarcoma Group (OSG),^[11,26]^ which makes a distinction between major and minor defects of tumor integrity. The KGSG survey showed that the results appeared to be similar to the definition of tumor rupture recently proposed by OSG; however, there are still some differences.^[10]^ Based on the classification of OSG, the 5-year RFS and OS were 88.5% and 91.6%, respectively, for patients with minor defects; and 93.6% and 94%, respectively, for patients with no defect of tumor integrity (*P* < .05) (Supplementary Fig. 1A and B); therefore, we adopted KGSG's definition in this study. Using a forward stepwise Cox model, our study found that the KGSG definition of rupture was an independent prognostic factor for both RFS and OS.

An initial multicenter clinical study (B2222) certified the clinical efficacy of IM in the treatment of patients with locally advanced or metastatic GIST, which provided a new tool in targeted therapy for patients with GIST.^[27]^ Then, a randomized double-blind phase III study (ACOSOG Z9001) showed that a 1-year duration of receiving IM therapy after surgery can improve patients’ RFS.^[12]^ Furthermore, the results from the Nordic III randomized study (SSG XVIII/AIO) suggested that postoperative oral IM treatment for 3 years can significantly improve the 5-year RFS rates and OS rates for patients with a high recurrence risk.^[28]^ Therefore, based on these studies, the NCCN guidelines recommended that patients with moderate risk should receive IM for at least 1 year, and at least 3 years of IM treatment is recommended for patients at high risk.

However, the appropriate duration of receiving postoperative adjuvant IM treatment remains controversial. In the study of ACOSCOG Z9001, the recurrence rate was 4% every year during the period of treatment, but it increased to 8% when IM treatment was stopped, while OS showed no obvious difference.^[29]^ Our previous study found that adjuvant IM for 3 years was not sufficient for high-risk patients with GISTs, and receiving 5 years of postoperative adjuvant IM therapy can significantly improve RFS of patients with high-risk GIST.^[17]^ Another retrospective study from a large volume institution supported that duration of adjuvant IM was the only favorable factor for outcomes of high-risk patients with GISTs.^[30]^

The optimal duration of adjuvant IM for GIST patients with tumor rupture who underwent resection has remained largely unknown and should therefore be carefully investigated. The benefit of prolonged treatment is currently being explored in the SSG XXII trial (3 vs 5 years of adjuvant IM), but the definition of tumor rupture in this protocol is not as clear-cut as in the present study. In the present study, along with time of receiving IM <3 years, patients with tumor rupture, who were truly at high risk, showed a 5-year RFS of 29.1% and a 5-year OS of 42.4%. However, fortunately, the 5-year RFS and OS of patients with tumor rupture were significantly increased with increased time of taking IM; if patients with tumor rupture received IM for more than 3 years, the 5-year RFS and OS were both raised to 80.8%, which suggested that postoperative adjuvant IM treatment is effective for patients with tumor rupture. Therefore, since a duration of adjuvant IM for 1 to 3 years might not be enough for tumor rupture patients, attending physicians should advise patients suffering tumor rupture GISTs to prolong the duration of adjuvant IM. However, large sample, multicenter, randomized controlled trials are required to acknowledge our hypothesis.

We recognize that there are several limitations to this study. First, this single-center study is retrospective in nature and has a long inclusion time span, as well as a limited number of patients, especially in the events of recurrence and death, although similar to most studies on the rupture of GISTs. Second, the preoperative, spontaneous, and intraoperative iatrogenic ruptures were not subanalyzed, since there is no significant difference between pre- or intraoperative rupture.^[10]^ Third, the benefits of longer IM treatment need to be balanced with treatment-related toxicity; however, adverse effects were not investigated in this study. Fourth, tumor site and gene type likely influence tumor rupture, and the efficacy of IM in such subgroups warrants further research. Fifth, the IM treatment duration for nonrupture patients after R1 resections was unresolved in the present study. However, the well-defined tumor rupture and the long-term follow-up represent strengths of the present study.

## 5 Conclusion

To the best of our knowledge, this is the first study to verify KGSG criteria for tumor rupture stratification. To improve the survival of patients with ruptured GISTs, more prolonged (≥3 years) IM adjuvant therapy should be considered. However, this study was conducted in a retrospective manner at a single center, and a well-designed multicenter randomized controlled study with a large sample size is warranted in further investigations.

## Author contributions

Jun Lu, Yun Dai, Hua-Long Zheng, Jian-Wei Xie, and Chao-Hui Zheng contributed to the study design, literature search, collection of the data, and data analysis. Jun Lu and Yun Dai contributed to the literature search and the writing of the manuscript. Jia-Bin Wang, Jian-Xian Lin, Qi-Yue Chen, Long-Long Cao, Mi Lin, Ru-Hong Tu, Ze-Ning Huang, Ju-Li Lin, Ping Li, and Chang-Ming Huang contributed to the review and revise of the manuscript.

**Conceptualization:** Jun Lu, Chao-Hui Zheng.

**Data curation:** Jun Lu, Yun Dai, Hua-Long Zheng, Chao-Hui Zheng.

**Formal analysis:** Jun Lu, Yun Dai, Hua-Long Zheng, Jian-Wei Xie.

**Funding acquisition:** Jun Lu, Chao-Hui Zheng.

**Investigation:** Hua-Long Zheng, Jian-Wei Xie, Chang-Ming Huang, Chao-Hui Zheng.

**Methodology:** Jun Lu, Yun Dai, Hua-Long Zheng, Jian-Wei Xie, Jia-Bin Wang.

**Project administration:** Yun Dai, Hua-Long Zheng, Jian-Wei Xie, Jia-Bin Wang, Long-Long Cao, Ping Li.

**Resources:** Jia-Bin Wang, Jian-Xian Lin, Qi-Yue Chen, Mi Lin, Ping Li.

**Software:** Yun Dai, Jian-Xian Lin, Qi-Yue Chen, Long-Long Cao, Mi Lin, Ru-Hong Tu, Ze-Ning Huang, Ju-Li Lin.

**Supervision:** Yun Dai, Jian-Wei Xie, Jia-Bin Wang, Jian-Xian Lin, Qi-Yue Chen, Long-Long Cao, Mi Lin, Ru-Hong Tu, Ze-Ning Huang, Ju-Li Lin, Ping Li, Chang-Ming Huang, Chao-Hui Zheng.

**Validation:** Jian-Wei Xie, Jia-Bin Wang, Jian-Xian Lin, Qi-Yue Chen, Long-Long Cao, Mi Lin, Ru-Hong Tu, Ze-Ning Huang, Ju-Li Lin, Ping Li, Chao-Hui Zheng.

**Visualization:** Yun Dai, Jian-Wei Xie, Jia-Bin Wang, Chang-Ming Huang, Chao-Hui Zheng.

**Writing – original draft:** Jun Lu, Yun Dai, Hua-Long Zheng.

**Writing – review & editing:** Jun Lu, Ping Li, Chang-Ming Huang, Chao-Hui Zheng.

## Supplementary Material

Supplemental Digital Content
