# Supplementary material for: What is the appropriate duration of adjuvant imatinib mesylate treatment for primary gastrointestinal stromal tumors classified according to the strict definition of tumor rupture?
Source: Medicine (Baltimore). 2019 Jan 18;98(3):e14177. doi: 10.1097/MD.0000000000014177 (PMC6370173; doi:10.1097/MD.0000000000014177)
Supplement: Supplemental Digital Content [file medi-98-e14177-s001.doc]

Supplementary Fig. 1 Recurrence-free (a) and overall survival (b) after resection of GISTs in relation to tumor integrity. Tumor integrity was defined by OSG.


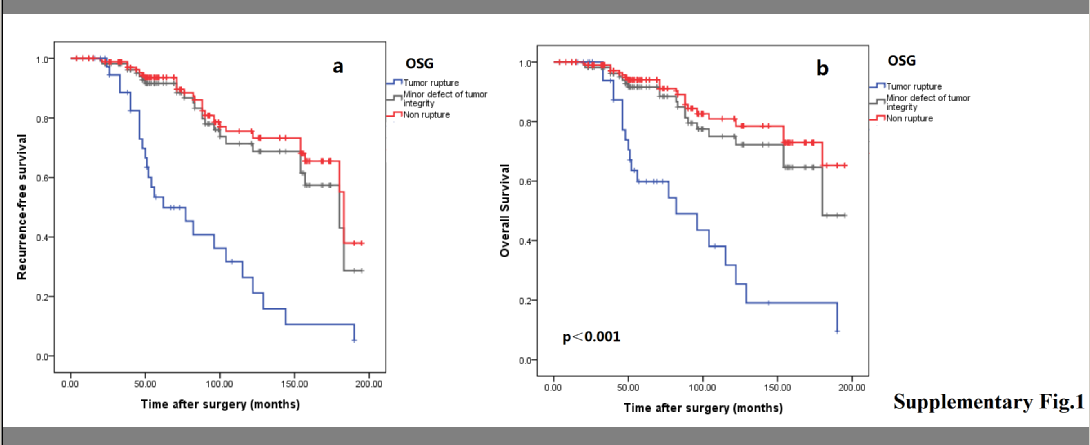


Supplementary table 1. Summary of series of studies on long-term outcomes in patients with and without tumor rupture

| Reference | Year | Country | No. of rupture | 5-yr  RFS(%)**^¶^**  Median RFS**^§^**  5-yr Recurren-ce rate ^♪^ | 5-yr OS(%)**^¶^**  Median RFS**^§^**  5-yr Recurren-ce rate ^♪^ | No. of Non-rupture | 5-yr RFS(%)**^¶^**  Median RFS**^§^**  5-yr Recurren-ce rate ^♪^ | 5-yr OS(%)**^¶^**  Median RFS**^§^**  5-yr Recurren-ce rate ^♪^ |
| --- | --- | --- | --- | --- | --- | --- | --- | --- |
| Yanagimoto et al. [1] | 2014 | Japan | 14 | NA | NA | 695 | NA | NA |
| Hohenberger et al. [2] |  |  |  |  |  |  |  |  |
| Joensuu et al. [3] | 2014 | Finland | 73 | 37.7**^¶^** | NA | 285 | 64.9**^¶^** | NA |
| Joensuu H et al. [4] | 2012 | Finland | 71 | NA | NA | 1127 | NA | NA |
| Rutkowski et al. [5] | 2011 | Finland | 46 | 17.2**^¶^** | NA | 594 | 54.7**^¶^** | NA |
| Wozniak et al. [8] | 2014 | Belgium | 54 | NA | NA | 800 | NA | NA |
| Nishida et al. [10] | 2018 | Japan | 21 | 2.4 years**^§^** | 6.4 years**^§^** | 644 | 8.4 years**^§^** | 11.9 years**^§^** |
| Holmebakk et al. [11] | 2018 | Norway | 22 | 37**^¶^** | NA | 242 | 96**^¶^** | NA |
| Hølmebakk et al. [27] | 2015 | Norway | 20 | 64^♪^ | NA | 31 | 31^♪^ | NA |

NA=not available, RFS=recurrence-free survival, OS=overall survival

**Supplementary table 2. Definition of tumor rupture according to KGSG and OSG criteria [10,26]**

| Defined by KGSG | Defined by OSG |
| --- | --- |
| **Tumor rupture** | **Tumor rupture** |
| Fracture with blood-tinged ascites | Spillage or fracture |
| Piecemeal resection | Piecemeal resection |
| Perforation at tumor site | Perforation at tumor site |
| Surgical biopsy | Surgical biopsy |
| Macroscopic injuries to the pseudocapsule | Adjacent organ infiltration |
| More than one of the above | Blood-tinged ascites |
| / | More than one of the above |
| / | **Minor defect of tumor integrity** |
| / | Peritoneal tumor penetration *(corresponding to a T4a*  *tumor in gastric carcinomas)* |
| / | Iatrogenic peritoneal laceration |
| / | Microscopically involved resection margins |
| / | More than one of the above |

KGSG=Kinki GIST Study Group, OSG=Oslo Sarcoma Group
